# A multicomponent intervention program to Prevent and Reduce AgItation and phySical rEstraint use in the ICU (PRAISE): study protocol for a multicenter, stepped-wedge, cluster randomized controlled trial

**DOI:** 10.1186/s13063-023-07807-x

**Published:** 2023-12-11

**Authors:** Rens W. J. Kooken, Bram Tilburgs, Rob ter Heine, Bart Ramakers, Mark van den Boogaard, on behalf of the PRAISE study group, Dieke Wiggelo-Lijbers, Margreet Klop-Riehl, Thijs C.D. Rettig, JanWillem Wijnhoven, Bram Simons, Rens van de Weyer, Stephanie Bakker, Wouter de Ruijter, Stefanie Slot, Stefanie Braker-Scholtes, Liz Bruin, Quirine Habes, Sanne Meeuws, Manon Fleuren-Janssen

**Affiliations:** 1 Department of Intensive Care Medicine, Radboud university medical center, Nijmegen, The Netherlands; 2 Department of Pharmacy, Radboud university medical center, Nijmegen, The Netherlands

**Keywords:** ICU, Agitation, Physical restraints, Non-pharmacological interventions, Dexmedetomidine, Patient-reported outcome measures

## Abstract

**Background:**

Physical restraints remain to be commonly used in agitated intensive care unit (ICU) patients worldwide, despite a lack of evidence on efficacy and safety and reports of detrimental short and long-term consequences, such as prolonged delirium and a longer ICU length of stay. Physical restraint minimization approaches have focused mainly on educational strategies and other non-pharmacological interventions. Combining these interventions with goal-directed light sedation therapy if needed may play an important contributory role in further reducing the use of physical restraints. The aim of the study is to determine the effectiveness of a multicomponent intervention (MCI) program, combining person-centered non-pharmacological interventions with goal-directed light sedation, compared to physical restraints.

**Methods:**

A multicenter stepped-wedge cluster randomized controlled trial will be conducted in six Dutch ICUs. A power calculation based total of 480 (expected to become) agitated adult patients will be included in 26 months with a subsequent 2-year follow-up. Patients included in the control period will receive standard care with the current agitation management protocol including physical restraints. Patients included in the intervention period will be treated with the MCI program, consisting of four components, without physical restraints: education of ICU professionals, identification of patients at risk for agitation, formulation of a multidisciplinary person-centered care plan including non-pharmacological and medical interventions, and protocolized goal-directed light sedation using dexmedetomidine. Primary outcome is the number of days alive and outside of the ICU within 28 days after ICU admission. Secondary outcomes include length of hospital stay; 3-, 12-, and 24-month post-ICU quality of life; physical (fatigue, frailty, new physical problems), mental (anxiety, depression, and post-traumatic stress disorder), and cognitive health; and 1-year cost-effectiveness. A process evaluation will be conducted.

**Discussion:**

This will be the first multicenter randomized controlled trial determining the effect of a combination of non-pharmacological interventions and light sedation using dexmedetomidine compared to physical restraints in agitated ICU patients. The results of this study, including long-term patient-centered outcomes, will provide relevant insights to aid ICU professionals in the management of agitated patients.

**Trial registration:**

NCT05783505, registration date 23 March 2023.

**Supplementary Information:**

The online version contains supplementary material available at 10.1186/s13063-023-07807-x.

## Background

The use of physical restraints (PRs) in the intensive care unit (ICU) remains a controversial topic. Although the use of PRs is increasingly considered inhumane and outdated [1, 2], the worldwide prevalence still varies widely from 0 to 75% [3, 4]. In the Netherlands, approximately 20–25% of patients are physically restrained during their ICU stay [5]. PRs are applied to ensure safety in patients who are (expected to become) agitated and as a result are at risk of falling or self-removing medical devices, such as tubes, catheters, and drains [3, 6–8]. Interestingly, however, several studies have shown an even greater incidence of adverse events that PRs intend to prevent, e.g., more unintentional IV-line removals, unplanned extubations, and reintubations [3, 9–12]. Moreover, the use of PRs is associated with skin and peripheral nerve injury, increased agitation, prolonged delirium duration, enhanced medication usage, and a longer ICU-length of stay (LOS), leading to higher healthcare costs [3, 4, 9, 13–15]. Besides the fact that PRs are considered humiliating by patients and their relatives, leading to further loss of autonomy and reduction of quality of life [9, 16, 17], PRs are also associated with detrimental psychological consequences in the long term. ICU patients in whom PRs were applied report more symptoms of anxiety and depression and are substantially more prone to develop post-traumatic stress disorder (PTSD) [4, 13, 18]. Hence, minimization of ICU PR use is a key priority for the Dutch ICU patient organization. However, despite the lack of evidence on efficacy and safety, as well as ethical considerations, the use of PRs is still part of daily ICU practice because of a presumed lack of safe alternatives [3, 4].

The initial step in caring for an agitated patient should prioritize a person-centered approach that focuses on non-pharmacological interventions (e.g., reduction of fear, stress, sleep deprivation and noise, and providing family presence), which can be complemented with goal-directed light sedation if needed. This integrated approach seems far more patient-friendly as compared to using PRs, likely improving patient outcomes [1, 19, 20]. Dexmedetomidine, a high-affinity α2-agonist inducing light sedation while preserving (a degree of) consciousness, is currently being advised as a sedative in mechanically ventilated patients [3]. Compared to other, more traditionally used sedatives in the ICU (e.g., propofol and midazolam), the use of dexmedetomidine results in reduced agitation, reduced delirium occurrence, less need for physical restraints, less coma days, a shorter time to extubation, and a shorter ICU-LOS [19, 21–24]. Moreover, and importantly, patients retain the ability to communicate. Therefore, dexmedetomidine seems an ideal sedative for agitated patients, especially since its use is already implemented in most ICUs [25]. Studies evaluating non-pharmacological interventions combined with goal-directed light sedation therapy using dexmedetomidine, however, are lacking. Therefore, the aim of this study is to determine the effectiveness of a person-centered multicomponent intervention (MCI) program, including non-pharmacological interventions and goal-directed light sedation using dexmedetomidine, on short- and long-term outcomes and healthcare costs, compared to the current standard of care including the use of physical restraints, in adult ICU patients who are (expected to become) agitated.

## Methods

### Study design and setting

This study protocol was written according to the SPIRIT guidelines (Additional file 1) [26]. The PRAISE study, a multicenter stepped-wedge cluster randomized controlled trial, will be carried out in six (non)-academic Dutch ICUs (www.clinicaltrials.gov: NCT05783505). The PRAISE study is part of the MONITOR-IC study (www.clinicaltrials.gov: NCT03246334), an ongoing multicenter prospective cohort study measuring long-term outcomes of ICU survivors [27].

**Fig. 1:**
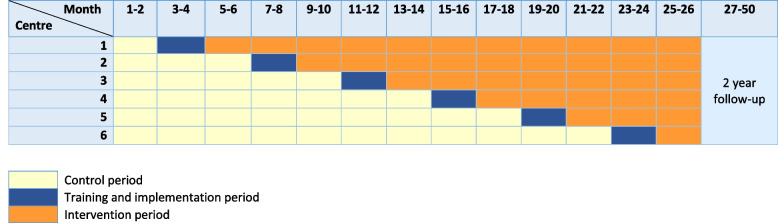
MCI program implementation sequence

A cluster-randomized design was chosen for the PRAISE study, since the MCI program is set to become the standard of care for all patients during the intervention period. Additionally, the in-depth training provided to ICU nurses and physicians (ICU professionals) during the implementation period could lead to a carry-over effect. As a result, randomization is only possible at the cluster (ICU) level. At the start, all participating ICUs include patients who receive the then-current standard of care, using PRs when necessary, serving as control patients (Fig. 1). Subsequently, ICU professionals will be extensively trained in the MCI program as part of a 2-month implementation period, whereafter the ICU enters the intervention period. An independent investigator will randomize the order in which an ICU starts with the intervention period by shuffling sealed envelopes. Every 4 months, the MCI program will be implemented in an additional ICU. From the intervention period onwards, the MCI program will become the new standard of care in the ICUs, and all included patients will serve as intervention patients. The planned inclusion period will be 26 months, with a subsequent 2-year follow-up. Every ICU will participate for the entire study period. The 2-month implementation period will be considered as a wash out period in which no patients will be included.

### Study population and eligibility criteria

All patients aged ≥ 18 years with an expected ICU stay of > 24 h, who are (expected to become) agitated within the first 14 days of their ICU admission, are eligible. Agitation will be defined as a Richmond Agitation and Sedation Scale (RASS) of ≥ 2 [28, 29], and expected agitation will be based on clinical judgment by the ICU professional.

Patients who meet any of the following criteria are not eligible for study participation:

- Contraindication for dexmedetomidine use (e.g., AV-block grade 2 or 3 unless a pacemaker is present, uncontrolled hypotension, acute cerebrovascular condition or known/suspected hypersensitivity);
- Neurological patients with an (expected risk of) increased intracranial pressure;
- An intoxication as a result of drug abuse (e.g., ethanol, γ-Hydroxybutyrate, opioids, benzodiazepines);
- Support with extracorporeal membrane oxygenation (ECMO);
- Difficult airway (as defined by the American Society of Anesthesiologists) [30];
- A high risk of physical aggression towards healthcare professionals;
- No consent for long-term follow-up in the MONITOR-IC study;
- Not able to read or understand the Dutch language and no relatives able to assist;
- Enrolment in other sedation studies.

### Sample size calculation

The sample size calculation is based on the study of Francken et al. [13], and all data were log-transformed for the purpose of sample size calculation. In the study, physically restrained ICU patients (*n* = 341) had a mean ICU-LOS of 6.27 days, while non-restrained patients (*n* = 1825) had a mean ICU-LOS of 2.53 days. Since data on sedation usage was not available, which could have affected ICU-LOS, a more conservative ICU-LOS of 4.27 days for non-restrained patients was used. To detect a difference of 2 days on ICU-LOS between restrained vs. non-restrained patients with a power > 80%, an intraclass correlation coefficient of 0.10, and *α* of 0.05, a total of 432 patients are needed. Anticipating an attrition loss of 10%, a total of 480 patients will be included, amounting to 3–4 patients per center per month (480 patients divided by 6 centers in 24 months).

### Control period: standard ICU care

During the control period, patients will receive the then-current standard ICU care, meaning that patients who are (expected to become) agitated will receive PRs if necessary. Patients can be enrolled if they are physically restrained during the first 14 days of their ICU admission. According to Dutch guidelines, physical restraint will be defined as any object or material that is attached to the body of the patient and to the bed or chair, with the purpose of limiting the patients’ freedom of movement (e.g., ankle- or wristbands, upper torso restraints) [31]. In case restrained patients are still agitated or at risk of falling or removing medical devices, ICU professionals can decide to administer a sedative (e.g., dexmedetomidine/clonidine, propofol or midazolam) as described in the current agitation protocols. Both PRs and the sedatives will be maintained as long as clinically deemed necessary.

### Intervention period: multicomponent intervention program

**Fig. 2:**
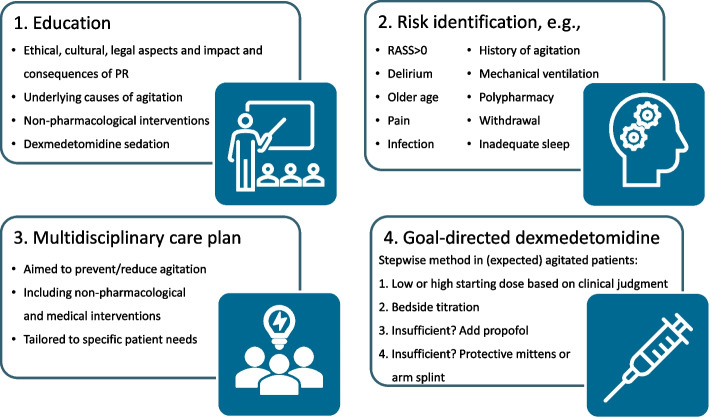
Multicomponent intervention program

The MCI program (Fig. 2 and Additional file 2) was developed and will be implemented using the Medical Research Council framework for developing complex interventions [32]. Close attention was given to all relevant core elements, for example the early involvement of stakeholders (former patients, ICU nurses and physicians), which has shown to be an important motivator for successful development and implementation of interventions [33]. The MCI program will be tailored specifically to each center and the patients’ needs, and consists of four components, without the use of physical restraints:

1. Education of ICU nurses and physicians, encompassing both interactive didactic and clinical-reasoning-based teaching methods, focusing on ethical, social, and legal considerations of physically restraining patients; impact and hazards of physical restraints; underlying causes of agitation; non-pharmacological interventions to prevent or reduce agitation; and goal-directed light sedation using dexmedetomidine.
2. Identification of patients who are (at risk to become) agitated through assessing patients’ level of consciousness, presence of delirium, and identifying other risk factors for agitation.
3. Formulation of a multidisciplinary person-centered care plan aiming to prevent or reduce agitation, including both evidence based non-pharmacological interventions as well as therapeutic interventions aimed at the medical domain (Additional file 2).
4. Protocolized goal-directed person-centered light sedation using dexmedetomidine (Additional file 2). In case the desired sedation level is not yet achieved upon reaching the maximum dose of dexmedetomidine (1 μg/kg/h), propofol will be administered simultaneously. If the co-administration of both sedatives is still insufficient to avert the agitation, as a last resort, special protective mittens or arm splints can be used, which still allow the patient’s freedom of movement (Additional file 2).

#### Implementation strategy and feasibility

In order to facilitate implementation, an expert group of dedicated ICU nurses, researchers, physicians, and (if possible) former patients will be composed in each participating center. In collaboration with these expert groups, barriers and facilitators related to existing routines as well as specific cultural elements and the organizational, educational, and practical conditions needed for implementation in each center will be determined [34, 35]. These barriers, facilitators, and conditions will be used to specifically tailor the educational and implementation strategies to each center. Moreover, members of the expert group will act as innovators and early adopters to motivate and stimulate (pre)contemplators to put the intervention into practice [36, 37]. The interventions will neither be applied nor communicated to other ICU professionals by any of the expert group members during the control period.

### Outcome measures and data collection

A schedule of enrolment, interventions, and assessments of this trial is shown in SPIRIT format in Table 1 [26]. The primary outcome is ICU-free days within 28 days after ICU admission, defined as the number of days alive and out of the ICU.

**Table 1:**
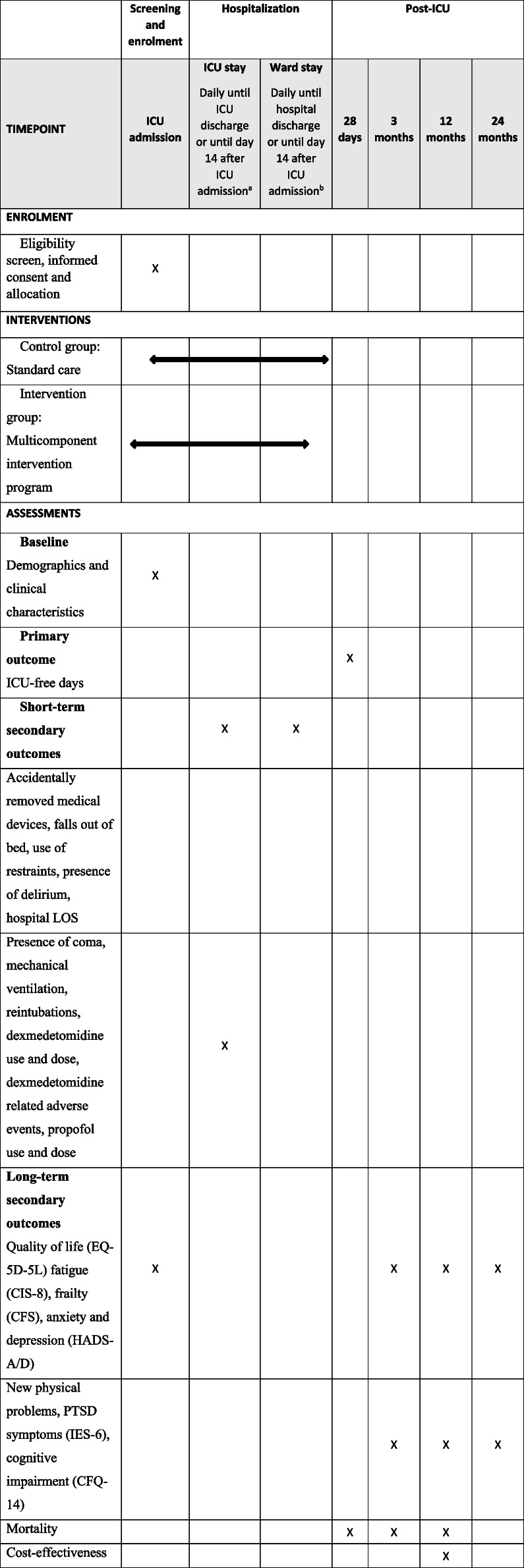
Schedule of enrolment, interventions, and assessments in SPIRIT format [26]

^a^Whichever comes first

^b^Only if discharged to the ward with delirium (defined as either a positive delirium assessment or treatment for delirium in the last 24 h)

Short-term secondary outcomes, collected for 14 days after ICU admission, include the following: incidence rate of accidentally removed medical devices (tubes, lines, drains, catheters) and falls out of bed; incidence rate of (self-extubation induced) reintubations; days with delirium; days with coma; number of delirium- and coma-free days; days with physical restraints or last-resort protective mittens/arm splints; days with dexmedetomidine and total administered dose; dexmedetomidine related adverse events (e.g., hypotension, bradycardia) that required intervention; and days with propofol and total administered dose. Patients who are discharged to the ward while delirious (defined as either a positive delirium assessment or treatment for delirium in the last 24 h) will be followed during ward stay until the end of the study period (i.e., 14 days after ICU admission) or hospital discharge, whichever comes first. Patients without delirium at the time of ICU discharge will be considered free from delirium and restraints and without accidental device removals until the end of the study period. Other short-term secondary outcomes include days on mechanical ventilator, hospital-LOS, and 28 day-mortality.

Long-term outcomes will be collected in the context of the MONITOR-IC study. The primary long-term outcome is 1-year post-ICU PTSD. Other long-term outcomes include patient-reported quality of life; physical (fatigue, frailty, new physical problems), mental (anxiety, depression, and PTSD), and cognitive health; and mortality at 3, 12, and 24 months post-ICU and 1-year cost-effectiveness.

#### Patient demographics and clinical variables

Patient demographics (e.g., age, gender), baseline clinical variables (e.g., admission type, severity of illness score), and short-term outcome measures (e.g., ICU-LOS, incidence rate of accidentally removed medical devices, days with dexmedetomidine and total administered dose) will be extracted from the Dutch National Intensive Care Evaluation (NICE) registry [38] and the patients’ electronic health records.

#### Delirium and level of consciousness

Presence of delirium will be assessed three times daily using a validated delirium screening instrument, i.e., the Intensive Care Delirium Screening Checklist (ICDSC; an 8-item checklist where a score ≥ 4 indicates delirium) or Confusion Assessment Method for the ICU (CAM-ICU; a 4-item scoring system where presence of the first two items and at least one of the latter two items indicates delirium) or the Delirium Observation Screening scale for the ward (DOS; a 13-item checklist where a score of ≥ 3 indicates delirium) [39–41]. A delirium day will be defined as a day with at least one ICDSC/CAM-ICU/DOS assessment above the cut-off for delirium.

Level of consciousness and agitation will be assessed with the Richmond Agitation and Sedation (RASS) score and noted on a scale from + 4 (combative) to − 5 (unarousable) [28, 29]. A day with coma will be defined as a day with at least one RASS score of − 4 or − 5. A day without either delirium or coma will be considered a delirium- and coma-free day. The screening for delirium and level of consciousness or agitation is part of standard daily practice in all Dutch ICUs.

#### Patient-reported outcomes

The following patient-reported outcomes will be collected (Table 2): quality of life (EuroQol 5 dimensional 5-level (EQ5D-5L) [42]), level of fatigue (Checklist Individual Strength (CIS-8) [43]), frailty (Clinical Frailty Scale (CFS) [44]), new or worsened physical problems after ICU admission [45], symptoms of anxiety and depression (Hospital Anxiety and Depression Scale (HADS) [46]), presence of PTSD symptoms (Impact of Event scale (IES)-6 [47]), and cognitive impairment (Cognitive Failure Questionnaire (CFQ) [48]).

**Table 2:** Overview of questionnaire items and scoring

Domain	Topic	Questionnaire	Items	Scoring and cut-off
**Quality of life**	Quality of life	EuroQol 5 dimensional 5-level (EQ-5D-5L) [42]	5 items (mobility, self-care, usual activities, pain/discomfort and anxiety/depression), with a 5-point Likert scale: “no problems” to “extreme problems/unable to” The EQ-5D-5L also includes a numeric scale in which participants rate their current overall health	A health state index value will be calculated from individual health profiles using the Dutch standard reference value set [49], with values ranging from − 0.446 (worse than death) to 1 (the value of full health) The numeric scale ranges from 0 (worst health imaginable) to 100 (best health imaginable)
**Physical health**	Fatigue	Checklist Individual Strength (CIS)-8 [43]	8 items with a 7-point Likert scale: “no, that is not right” (1) to “yes, that is right” (7)	The total score ranges from 8 to 56, where fatigue is indicated by a score of 27 or higher. A score of 27–35 indicates mild fatigue, a score higher than 35 indicates severe fatigue [50]
	Frailty	Clinical Frailty Scale (CFS) [44]	One-item scale on which a score of 1 indicates a very fit person, and a score of 9 indicates a terminally ill person	A score between 5 and 9 will be considered “frail” and a score between 1 and 4 will be considered “non-frail” [51]
	New physical problems	New physical problems after ICU admission [45]	30 items (i.e., pain, muscle weakness, loss of taste, shortness of breath) with a 4-point Likert scale: no problems, mild, moderate or severe problems	Answers will be dichotomized into “no problems” (no or mild problems) or “problems” (moderate or severe problems)
**Mental health**	Anxiety and depression symptoms	Anxiety and depression subscales of the Hospital Anxiety and Depression Scale (HADS) [46]	Both subscales consist of 7 items with a 4-point Likert scale ranging from 0 to 3	Each subscale has a total score ranging from 0 to 21. A score of 8 or higher on the anxiety or depression subscale indicates symptoms of anxiety or depression, respectively. A score of 8–10 indicates mild symptoms, 11–14 moderate symptoms, and 15–21 severe symptoms
	Post-traumatic stress disorder (PTSD) symptoms	Impact of Event Scale (IES)-6 [47]	6 items with a 5-point Likert scale: “not at all” (0) to “extremely” (4)	Total score is the mean of the six items, ranging from 0 to 4. A mean score of 1.75 or higher on all questions will be used as a cut-off score to differentiate between presence of PTSD symptoms or not [52]
**Cognitive health**	Cognitive impairment	Cognitive Failure Questionnaire (CFQ)-14 [48]	14 items with a 5-point Likert scale: “never” (0) to “very often” (4)	The total score ranges from 0 to 56. After transformation of the scores to a 0–100 range, cognitive impairment is indicated by a score of 43 or higher [53]

Except for new or worsened physical problems, PTSD symptoms and cognitive impairment, which will not be assessed at baseline, all patient-reported outcomes will be measured at baseline (ICU admission) and 3, 12, and 24 months after ICU admission (Table 1). All questionnaires are well validated, and part of the core outcome set for long-term outcomes of ICU survivors [54]. Depending on patient preferences, questionnaires can be completed online, by paper or by telephone. Non-responders will be reminded after 4 and 6 weeks.

### Patient recruitment and timeline

**Fig. 3:**
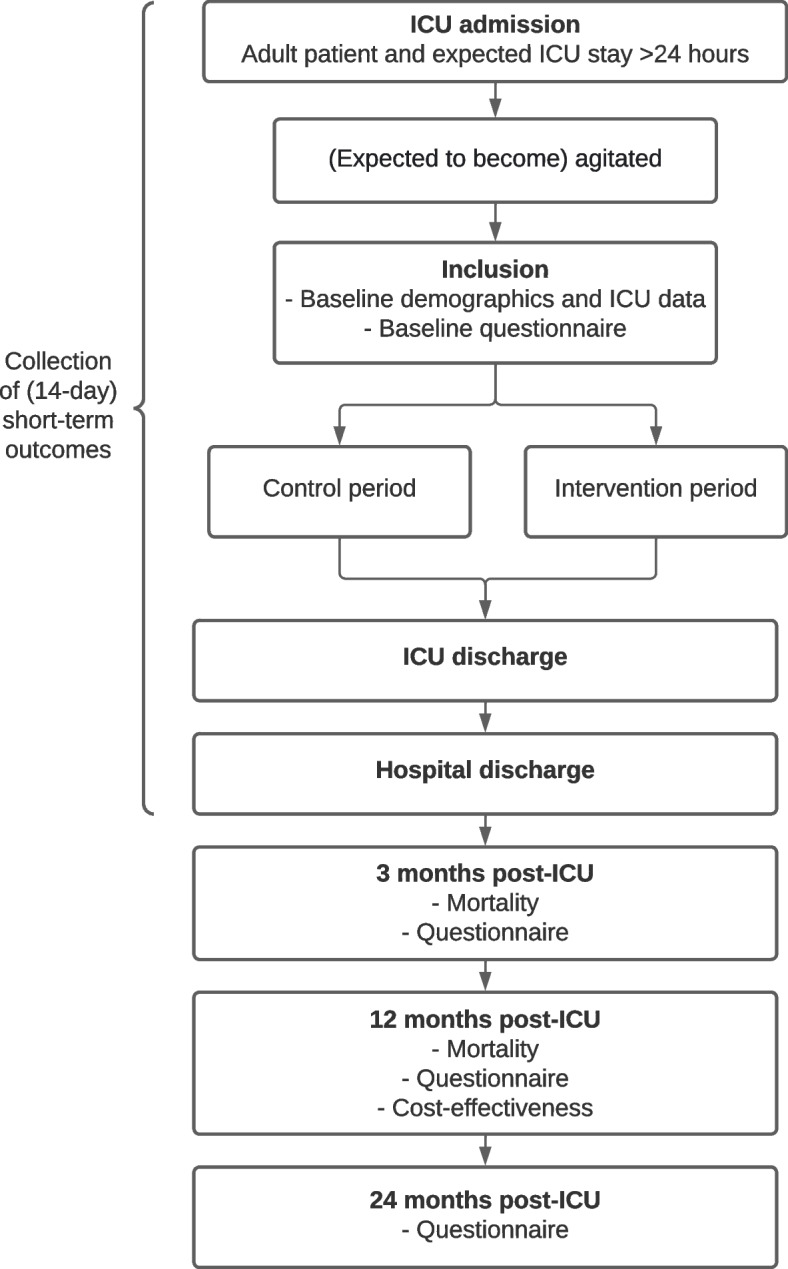
Study flowchart of patient inclusion and data collection. ICU, intensive care unit

A (research) nurse, physician, or researcher will inform eligible patients and/or their relatives and ask them to participate in the study (Fig. 3). In case consent cannot be given directly (e.g., emergency admission, sedated patient with no family present), baseline data will be collected retrospectively after consent has been obtained. To note, regardless of consent, all patients will be treated using the MCI program in the intervention period, as it will be the new standard of care in all participating hospitals.

### Data analysis

Analyses will follow the intention-to-treat principle. Secondary per-protocol analyses will also be conducted, excluding patients who were not treated according to protocol (e.g., use of PR in the intervention period).

Descriptive statistics will be used to describe demographics and baseline characteristics. Linear and logistic multi-level models will be used to compare outcomes between the intervention and control group. A multilevel Cox proportional hazards model will be used for time-to-event outcomes. Crude and adjusted analyses (e.g., center effect) will be performed. Heterogeneity of treatment effect will be explored in different subgroups, e.g., gender, age, presence of delirium, duration of treatment, and use of last-resort mittens or splints. Missing value analyses will be conducted. Missing data will be handled according to questionnaire manuals or imputed using multiple imputation methods if necessary. The economic evaluation will follow the principles of a cost-utility analysis (cost per quality-adjusted life year (QALY) gained, using EQ-5D-5L as outcome measure) as well as a cost-effectiveness analysis (outcome 1 year PTSD) and adheres to the Dutch guideline for performing economic evaluations in health care [55].

#### Process evaluation

A comprehensive process evaluation will be conducted in accordance with international guidelines, in which barriers and facilitators and other contextual factors influencing implementation of the intervention program will be explored [32, 56]. The process evaluation will include qualitative methods, i.e., focus group interviews with relevant stakeholders such as nurses and physicians, as well as quantitative methods, i.e., data on inclusion rates, use of PRs in the intervention period, and questionnaires to evaluate the education program and intervention adherence.

## Discussion

Despite numerous reports on the deleterious consequences and ineffectiveness of physical restraints, a subjective lack of safe alternatives still withholds ICU professionals worldwide from abandoning its use. Moreover, only a few randomized controlled trials have been conducted on physical restraint minimization strategies in the ICU, focusing solely on educational and other non-pharmacological interventions [57]. Although these interventions should always remain as the first step in mitigating the need for restraining therapies, the use of goal-directed light sedation may play an important contributory role in further reducing PR use, especially in light of the promising results of dexmedetomidine. This will be the first randomized controlled trial worldwide investigating the combination of non-pharmacological interventions and light sedation using dexmedetomidine compared to standard care including physical restraints in agitated patients.

Due to medical advances and improved post-ICU care, more patients survive their critical illness, of whom many experience impairments in physical, cognitive, or mental health, generally described as post-intensive care syndrome (PICS) [45, 58, 59]. In light of the mounting evidence on the long-term impact of an ICU admission, the significance of patient-centered outcomes such as quality of life and PTSD is increasingly being acknowledged instead of short-term clinical outcomes like mortality [60]. This study’s findings will offer valuable insights into these long-term outcomes, both pre- and post-implementation of the MCI program, and will assist ICU professionals in making informed decisions regarding ICU agitation.

### Strengths and limitations of this study

- The main strength of this study is the multicenter stepped-wedge cluster randomized controlled design, with a long-term follow-up including patient-centered outcomes, a cost-effectiveness analysis, and a comprehensive process evaluation.
- The early engagement of relevant stakeholders in the development of the multicomponent intervention (MCI) program and the fact that the program will be tailored to the center and patient’s specific needs is another strength of this study, contributing to the improvement of person-centered ICU healthcare.
- The implementation of the new intervention program can pose a challenge, as ICU nurses and physicians (ICU professionals) need to let go of long-lasting old beliefs and contribute to a new restraint-free ICU culture.
- Collection of baseline and long-term outcomes using patient-reported outcome measures (PROMs) may introduce bias and is a study limitation, especially as part of the baseline questionnaires will be completed retrospectively (due to the acute nature of the ICU). However, in those cases, pre-admission health status will be evaluated as shortly as possible after admission. Moreover, proxies can help fill in questionnaires if the patient is unable to, limiting the extent of possible bias.

## Trial status

Recruitment is planned to take place from June 2023 up and until August 2025, with a subsequent 2-year follow-up. This paper is in accordance with the approved latest version of the protocol (January 2023).

### Supplementary Information

**Additional file 1.** SPIRIT checklist.

**Additional file 2.** MCI program.

**Additional file 3.** Consent form patient.

**Additional file 4.** Consent form legal representative.

**Additional file 5.** Ethical approval (translated).

**Additional file 6.** Funding documentation.

## Data Availability

Data will be collected, stored, and handled using Castor EDC and the MONITOR-IC database [ 61]. Data quality will be checked by at least two researchers of the study team. To maintain anonymity in the dataset, every participant will be identified with a unique six-digit study ID. The translation key will be saved on a secured database server of the Radboud University Medical Center and will only be accessible to RK, BT, and MvdB. Data will be securely stored for minimally 15 years. Data will be shared according to the FAIR principles upon reasonable request [ 62].

## Abbreviations

CAM: Confusion Assessment Method
DOS: Delirium Observation Screening
ICDSC: Intensive Care Delirium Screening Checklist
ICU: Intensive care unit
LOS: Length of stay
MCI: Multicomponent intervention
PICS: Post-intensive care syndrome
PR: Physical restraint
PROM: Patient-reported outcome measure
PTSD: Post-traumatic stress disorder
QALY: Quality-adjusted life year
QoL: Quality of life
RASS: Richmond Agitation and Sedation Scale
