# Supplementary material for: A multicomponent intervention program to Prevent and Reduce AgItation and phySical rEstraint use in the ICU (PRAISE): study protocol for a multicenter, stepped-wedge, cluster randomized controlled trial
Source: Trials. 2023 Dec 11;24:800. doi: 10.1186/s13063-023-07807-x (PMC10712112; doi:10.1186/s13063-023-07807-x)
Supplement: Supplementary file 2 — Additional file 2. MCI program. [file 13063_2023_7807_MOESM2_ESM.docx]

**Additional file 2. Multicomponent intervention (MCI) program**

In the intervention period, all patients who are (expected to become) agitated will be treated with the person-centered multicomponent intervention (MCI) program. The MCI program consists of four components:

1. Education of nurses and physicians (ICU professionals) on patient rights and autonomy, cultural, social and ethical aspects of restraining patients and impact and effects of physical restraint use on patients, including a video interview of a formerly restrained ICU patient. Furthermore, education on (underlying causes of) agitation, non-pharmacological interventions to prevent or reduce agitation, and person-centered goal-directed use of dexmedetomidine.
2. Identification of patients who are (at risk to become) agitated, through assessing patients’ level of consciousness (using the Richmond Agitation Sedation Scale (RASS) [1, 2]), presence of delirium (using the Intensive Care Delirium Screening Checklist (ICDSC) or Confusion Assessment Method (CAM-ICU) [3, 4]) and through identifying risk factors for agitation (e.g., delirium, older age, history of agitation, pain, infection/sepsis, electrolyte disturbances, mechanical ventilation, etc.). [5-7] Risk for agitation is defined as patients of whom ICU clinicians suspect are at risk for agitation, for example due to an ICDSC/CAM-ICU which is positive for delirium and/or presence of other risk factors. These patients will receive the further steps of the MCI program. [5]
3. Formulation of a multidisciplinary person-centered care plan (in joint consultation with nurses, physicians, the patient and the family) aiming to prevent or reduce agitation. This plan will include both non-pharmacological interventions as well as therapeutic interventions aimed at the medical domain (Table 1 and Figure 1).
4. Goal-directed light sedation therapy using dexmedetomidine (Figure 2), which will be combined with non-pharmacological and medical interventions (which will always be the foundation of agitation treatment) as formulated in the person-centered care plan.
   1. (Expected) agitated patients ((expected) RASS ≥2) will receive dexmedetomidine intravenously. At the discretion of the nurse or physician, starting dose may vary, depending on the situation. Already agitated patients will be started on the maximum dose (1 μg/kg/hour), while a lower range dose (0.4-0.6 μg/kg/hour) will be used in patients expected to become agitated. Doses will be titrated based on clinical effects until a guideline recommended target sedation level of RASS +1 to -2 and a minimalized risk of incidents is achieved, with a maximum dose of 1 μg/kg/hour. Sedation levels will be closely monitored and adjusted as appropriate according to protocol to minimize complications associated with over- and undersedation. [8] It may take up to one hour after dose titration before a new stable sedation level is reached. [9]

A concurrent symptomatic delirium (i.e., delirium with agitation, restlessness or hallucinations) will be treated according to protocol alongside dexmedetomidine infusion. In case of an adverse event (e.g., hypotension, bradycardia, allergic reaction) thought to be related to dexmedetomidine, dosage will be lowered or stopped according to local protocols.

- 1. If the required sedation level is not yet achieved after reaching the maximum dose of dexmedetomidine, propofol will be concurrently administered while maintaining the maximum dose of dexmedetomidine. Propofol doses will be titrated based on clinical judgment of the sedation level in consonance with center-specific guidelines.
  2. In case the conjoint use of dexmedetomidine and propofol is still insufficient to avert the agitation (i.e., RASS>1 after maximum dose of both sedatives), special protective mittens or arm splints will be used which still allow the patient’s freedom of movement (Figure 3 and 4).
  3. In case of an immediate threat to patient safety (e.g., near-extubation caused by agitation), the above steps will be skipped and a bolus injection of 10-20 mg propofol will be administered immediately, combined with a dexmedetomidine infusion of 1 μg/kg/hour.

The possibility to scale down or stop the pharmacological treatment will be evaluated every shift.

**Multidisciplinary person-centered care plan**

All interventions will be tailored specifically to the patient’s needs and preferences.

Table 1. Summary of non-pharmacologic interventions to prevent or reduce agitation

| Domain | Topic | Patient specific interventions | Detailed description |
| --- | --- | --- | --- |
| Sensory input | 1. Touch [10] | - Expressive touch | Use expressive touch to let patients know ICU staff is focused on them and to provide comfort. |
|  | 2. Vision [11-14] | - Prevent cornea dehydration during sedation |  |
|  |  | - Write a note in patient file in case of visual impairment | Write a clearly visible note in the electronic patient file in case of visual impairment and note what type of visual aid the patient uses. |
|  |  | - Use of visual aids | Ensure patient use of (clean) visual aids during the day. |
|  |  | - Approach patient from good vision side |  |
|  |  | - Use both verbal and non-verbal communication | Pay extra attention to the combination of verbal and non-verbal communication when executing (nursing) activities and describe which bedside you are at. |
|  |  | - Use specifically adapted material for visually impaired patients | Maximize communication by using material specifically adapted to visually impaired patients, like large-print books, audiobooks, tablets, and add fluorescent tape to call bell. |
|  | 3. Hearing [11, 12, 14] | - Speak clear and limit background noise |  |
|  |  | - Write a note in patient file in case of hearing impairment | Write a clearly visible note in the electronic patient file in case of hearing impairment and note what hearing aid the patient uses. |
|  |  | - Ask how to call the patient | Ask patient and/or family how to call the patient (e.g., first name, last name, nickname) |
|  |  | - Use of hearing aids | Ensure patient use of (functioning) hearing aids during the day. |
|  |  | - Remove hearing aids during rest | In consultation with the patient, remove the hearing aids during the night or moments of rest. |
|  |  | - Approach patient from good hearing side |  |
|  |  | - Use both verbal and non-verbal communication | Pay extra attention to the combination of verbal and non-verbal communication when executing (nursing) activities and describe which bedside you are at. |
|  |  | - Use special communication techniques | Maximize communication by using special communication techniques, like easy (hand)gestures, writing, pictures/symbols, letter boards, tablets. |
| Day-night rhythm | 1. Noise reduction at night [7, 11, 14-18] | - Unit wide noise reduction at night | Quiet hallways, closed doors, no loud talking, beepers or telephones on vibrate. Provide colleagues with noise feedback. |
|  |  | - Reduce alarm volumes and liberate alarm boundaries | Reduce alarm volumes and liberate alarm boundaries of the mechanical ventilator or monitor if possible. |
|  |  | - Apply earplugs during the night | In consultation with the patient, apply earplugs during night shift. |
|  | 2. Light reduction at night [7, 11, 14-16] | - Dim or turn off lights during the night | Dim or turn off lights in all patient and communal rooms during the night. |
|  |  | - Dim monitor screens and away from patient’s sight | Dim monitor screens and turn them away from the patient |
|  |  | - Keep patient room dark at night | Keep the patient room dark at night using curtains or lamellae. |
|  | 3. Sleep [7, 15-18] | - Discourage daytime sleeping | Discourage daytime sleeping, make use of a day schedule including all activities for that day |
|  |  | - Avoid procedures during sleep time | Avoid nursing and medical procedures during sleep time if possible. |
|  |  | - Apply earplugs during the night | In consultation with the patient, apply earplugs during nighttime. |
|  |  | - Address and acknowledge sleep problems if present. | Comfort patients who are unable to sleep, address their needs, let them know you are there for them. |
|  |  | - Room temperature | Pay attention to a pleasant room temperature during the night (consult patient) |
|  |  | - Use relaxation music to promote sleep | In consultation with the patient, provide relaxation music to promote sleep (make use of headphones). |
|  |  | - Consult family and patient for sleep techniques | Consult family and patient for patient-specific techniques to promote sleep |
|  |  | - Use of sleep medication | Only use sleep medication as a last resort (preference for short-acting non-benzodiazepine drugs such as zopiclone, zolpidem) |
| Orientation and distraction | 1. Orientation and communication [11-14, 19, 20] | - Communicate names of daily ICU staff and a day schedule | Place the names of the nurse, physician and a day schedule in the patient’s sight |
|  |  | - Clock and calendar | Place a clock and calendar in the patient’s sight |
|  |  | - Clear introduction to patient | Always introduce yourself to the patient with name, function and reason to be in the room |
|  |  | - Frequent information about hospital stay and current situation | At the beginning of each dayshift, provide the patient with simple and short information about the hospital stay and the patient’s current situation (i.e., hospital name, location, ICU, length of stay, reason for hospitalization, and illness progression) |
|  |  | - Ask or explain today’s time and date, location and reason for hospitalization | Stimulate the patient’s orientation as part of the daily routine by asking or explaining them what day and time it is (morning, afternoon, evening, night), where they are and why they are there. |
|  |  | - Be present in the patient’s room as much as possible and communicate with the patient. Provide comfort and support during periods of anxiety, fear or stress. | Explore what’s on the patient’s mind. Ask for their needs, perceptions, experiences, presence of fears, anxiety, hallucinations, and acknowledge them. Explain everything you do and the reason for doing it. |
|  |  | - Explain about delirium (if present) and acknowledge patient perceptions | In case of a delirium, explain about it. Inform the patients that confusion is commonly seen in severely ill patients. Acknowledge patient perceptions. |
|  |  | - Orientation to the window | Orient the patient’s bed to the window for perception of daylight/darkness |
|  |  | - Facilitate regular visits from friends and family |  |
|  |  | - Provide family with information on reorientation and presence of family/friends. | Provide the family with information on the importance of preventive measures like reorientation and the presence of family or friends. |
|  |  | - Stimulate family to reorient patient | Stimulate family to reorient patient (e.g., regarding current date and time, recent events, reason for hospitalization, etc). |
|  |  | - Familiar objects in the room | Bring familiar objects to the patient’s room, in consultation with the family and healthcare professionals. |
|  | 2. Distraction [11, 12, 14, 21-23] | - Provide distraction using TV | Provide distraction using television (consult patient/family for patient preferences). Use a headphone when a patient is watching television. |
|  |  | - Provide distraction using music | Provide distraction using music (consult patient/family for patient preferences). If no preferences, provide nature-based sounds or classical relaxation music. Use a headphone when a patient is listening to music. |
|  |  | - Provide distraction with familiar photographs | Provide distraction by showing familiar photographs/pictures (of the patient and his/her family/friends or other patient preferences). If no preferences, provide nature-based pictures. |
|  |  | - Provide distraction using (audio)book(s), magazine(s), newspaper(s) | Stimulate family to bring these materials. |
| (Early) mobilization [24-28] |  | - Frequently reposition immobile patients | Provide patient repositioning every three to four hours in immobile patients to prevent pressure ulcers. |
|  |  | - Pursue RASS 0 to permit (active) physical therapy |  |
|  |  | - Assess immobilizing equipment | Perform a daily assessment of the necessity of immobilizing equipment (e.g., tubes, lines, drains, catheters). |
|  |  | - Set multidisciplinary mobilization goals | Set multidisciplinary (nurse, physician, physical therapist) target mobilization goals and discuss timing. |
|  |  | - Provide physical exercises at least once daily | Provide physical exercises at least once daily (expand if possible). Physical exercises can range from passive, assisted-active or active range of motion exercises to sitting on edge of bed, standing at side of bed, walking to bedside chair, sitting in chair, marching in place or walking in room/unit. |
|  |  | - Stimulate doing frequent motion exercises | Encourage and remind patients to do motion exercises multiple times a day. Stimulate active participation during daily care activities. |
|  |  | - Temporarily disconnect immobilizing equipment for physical activity | Promote physical activity by temporarily disconnecting and capping immobilizing equipment (e.g., tubes, lines, drains and catheters) |
| Family participation [24, 29, 30] |  | - Ask family about the patient and his/her preferences | Invite family to provide information about the patient and his/her preferences (e.g., favorite music, television program, books, hobbies, and other things important to the patient) |
|  |  | - Invite family for bedside presence as much as possible | Invite family for bedside presence as much as possible. Consider no or minimal restrictions in visiting times. |
|  |  | - Provide family with information on reorientation and presence of family/friends | Provide the family with information on the importance of preventive measures like reorientation and the presence of family or friends. |
|  |  | - Stimulate family to reorient patients | Stimulate family to reorient patient (e.g., regarding current date and time, recent events, reason for hospitalization, etc). |
|  |  | - Provide an ICU diary | Provide an ICU diary for family members to write daily notes, for patient and family processing of the ICU stay. |
|  |  | - Write down important events in the ICU diary | ICU staff should also write down important events and happenings in the ICU diary, so that patients and family can reconstruct the ICU stay. |

**Therapeutic interventions aimed at the medical domain**


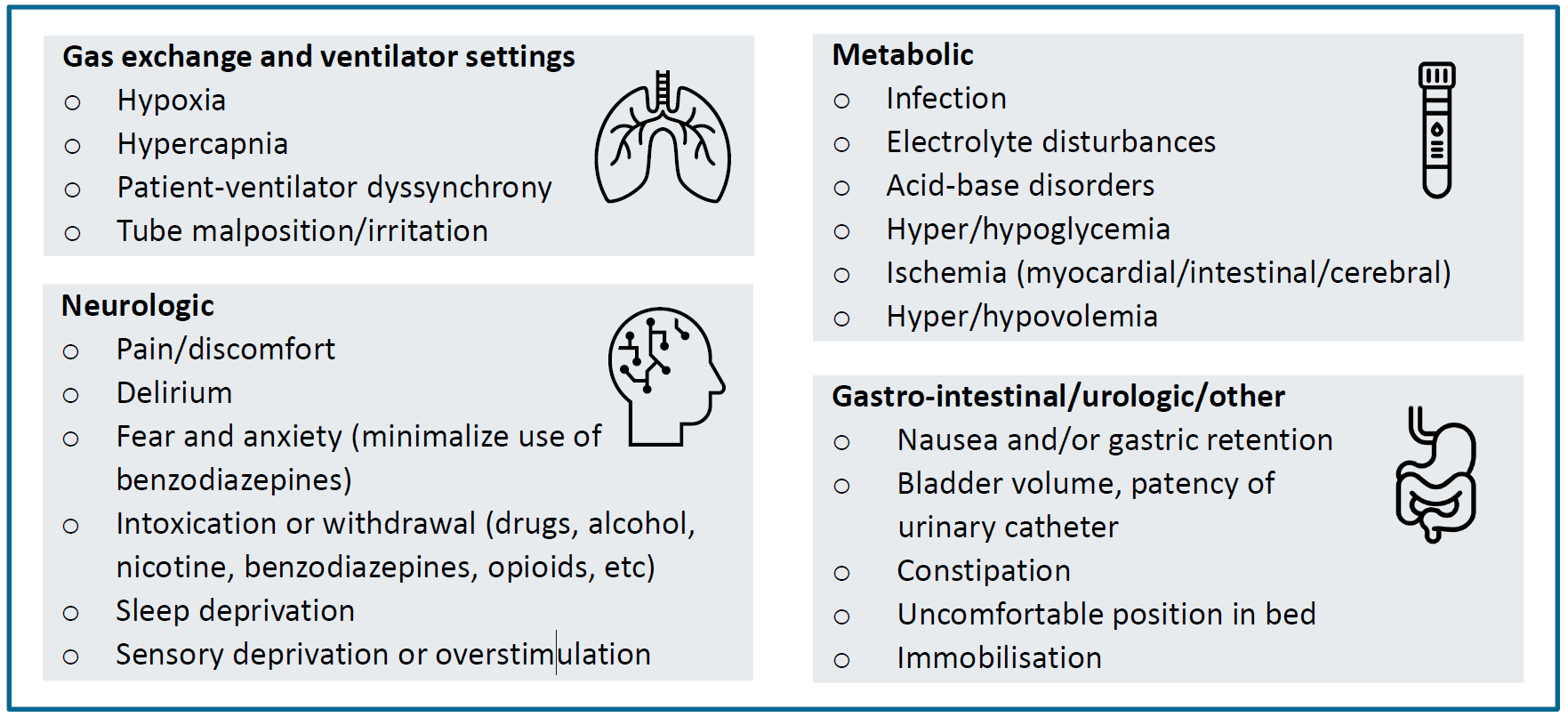


Figure 1. Medical treatment options for underlying causes of agitation [31-34]


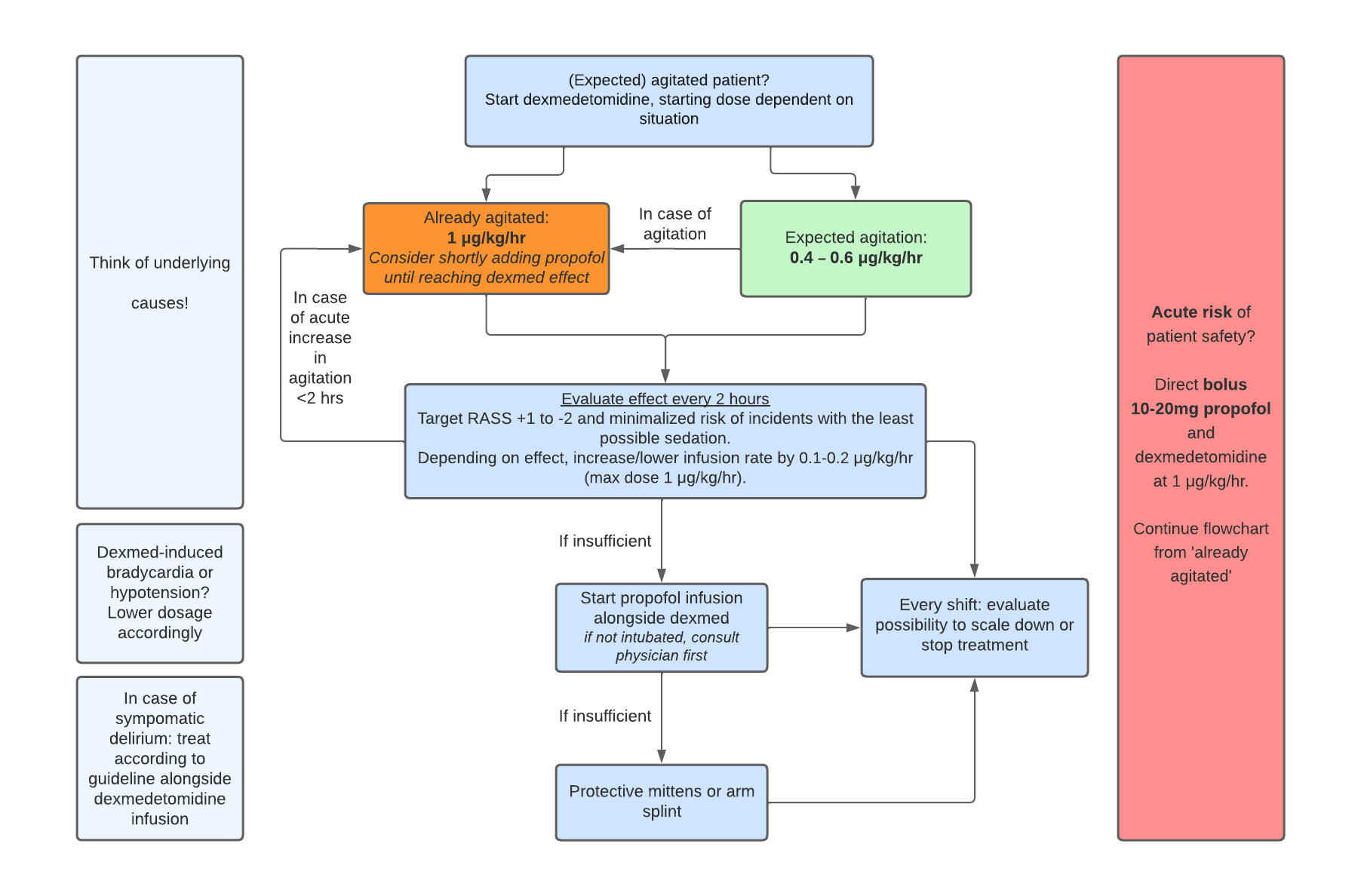


Figure 2. Dexmedetomidine dosing protocol


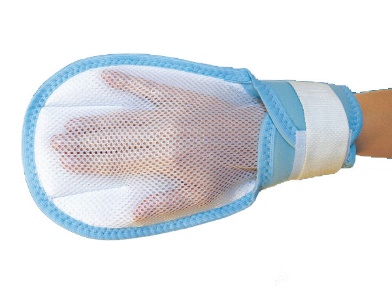


Figure 3. Protective mittens

**
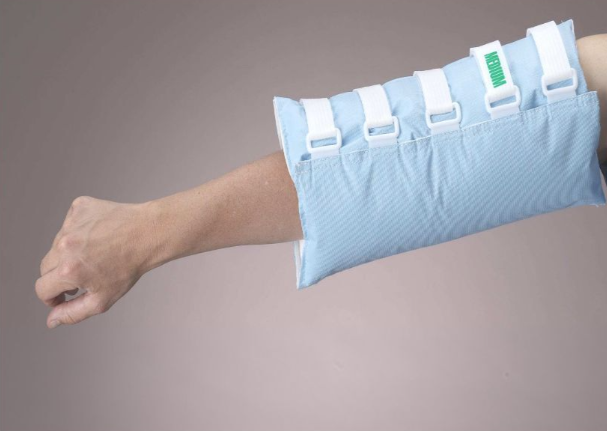
**

Figure 4. Arm splint

**References**

1. Sessler, C.N., et al., *The Richmond Agitation–Sedation Scale: validity and reliability in adult intensive care unit patients.* American journal of respiratory and critical care medicine, 2002. **166**(10): p. 1338-1344.

2. Ely, E.W., et al., *Monitoring sedation status over time in ICU patients: reliability and validity of the Richmond Agitation-Sedation Scale (RASS).* Jama, 2003. **289**(22): p. 2983-2991.

3. Khan, B.A., et al., *The Confusion Assessment Method for the ICU-7 Delirium Severity Scale: A Novel Delirium Severity Instrument for Use in the ICU.* Crit Care Med, 2017. **45**(5): p. 851-857.

4. Bergeron, N., et al., *Intensive Care Delirium Screening Checklist: evaluation of a new screening tool.* Intensive care medicine, 2001. **27**(5): p. 859-864.

5. Lach, H.W., K.M. Leach, and H.K. Butcher, *Evidence-Based Practice Guideline: Changing the Practice of Physical Restraint Use in Acute Care.* J Gerontol Nurs, 2016. **42**(2): p. 17-26.

6. Philabaum, A., *Physical Restraint Use in Adult Intensive Care Units (ICUs): A Systematic Review*, in *College of Nursing Honors*. 2016, The Ohio State university: Ohio.

7. Devlin, J.W., et al., *Clinical Practice Guidelines for the Prevention and Management of Pain, Agitation/Sedation, Delirium, Immobility, and Sleep Disruption in Adult Patients in the ICU.* Crit Care Med, 2018. **46**(9): p. e825-e873.

8. Jackson, D.L., et al., *The incidence of sub-optimal sedation in the ICU: a systematic review.* Critical Care, 2009. **13**(6): p. R204.

9. Corporation, O. *SmPC Dexdor*. 2018.

10. Papathanassoglou, E.D. and M.D. Mpouzika, *Interpersonal touch: physiological effects in critical care.* Biol Res Nurs, 2012. **14**(4): p. 431-43.

11. Inouye, S.K., et al., *A multicomponent intervention to prevent delirium in hospitalized older patients.* N Engl J Med, 1999. **340**(9): p. 669-76.

12. Young, J., et al., *Diagnosis, prevention, and management of delirium: summary of NICE guidance.* BMJ, 2010. **341**: p. c3704.

13. Martinez, F.T., et al., *Preventing delirium in an acute hospital using a non-pharmacological intervention.* Age Ageing, 2012. **41**(5): p. 629-34.

14. Rood, P.J.T., et al., *The Impact of Nursing Delirium Preventive Interventions in the ICU: A Multicenter Cluster-randomized Controlled Clinical Trial.* Am J Respir Crit Care Med, 2021. **204**(6): p. 682-691.

15. Luther, R. and A. McLeod, *The effect of chronotherapy on delirium in critical care–a systematic review.* Nursing in Critical Care, 2018. **23**(6): p. 283-290.

16. Kamdar, B.B., et al., *The effect of a quality improvement intervention on perceived sleep quality and cognition in a medical ICU.* Crit Care Med, 2013. **41**(3): p. 800-9.

17. Hu, R.F., et al., *Non-pharmacological interventions for sleep promotion in the intensive care unit.* Cochrane Database Syst Rev, 2015. **2015**(10): p. Cd008808.

18. Van Rompaey, B., et al., *The effect of earplugs during the night on the onset of delirium and sleep perception: a randomized controlled trial in intensive care patients.* Critical Care, 2012. **16**(3): p. R73.

19. Colombo, R., et al., *A reorientation strategy for reducing delirium in the critically ill. Results of an interventional study.* Minerva Anestesiol, 2012. **78**(9): p. 1026-33.

20. Marcantonio, E.R., et al., *Reducing delirium after hip fracture: a randomized trial.* J Am Geriatr Soc, 2001. **49**(5): p. 516-22.

21. Adams, A.M.N., et al., *Nonpharmacological interventions for agitation in the adult intensive care unit: A systematic review.* Aust Crit Care, 2022.

22. Saadatmand, V., et al., *Effect of nature-based sounds' intervention on agitation, anxiety, and stress in patients under mechanical ventilator support: a randomised controlled trial.* Int J Nurs Stud, 2013. **50**(7): p. 895-904.

23. Chlan, L.L., et al., *Effects of patient-directed music intervention on anxiety and sedative exposure in critically ill patients receiving mechanical ventilatory support: a randomized clinical trial.* Jama, 2013. **309**(22): p. 2335-44.

24. Pun, B.T., et al., *Caring for Critically Ill Patients with the ABCDEF Bundle: Results of the ICU Liberation Collaborative in Over 15,000 Adults.* Crit Care Med, 2019. **47**(1): p. 3-14.

25. Doiron, K.A., T.C. Hoffmann, and E.M. Beller, *Early intervention (mobilization or active exercise) for critically ill adults in the intensive care unit.* Cochrane Database Syst Rev, 2018. **3**(3): p. Cd010754.

26. Schweickert, W.D., et al., *Early physical and occupational therapy in mechanically ventilated, critically ill patients: a randomised controlled trial.* Lancet, 2009. **373**(9678): p. 1874-82.

27. Brummel, N.E., et al., *Feasibility and safety of early combined cognitive and physical therapy for critically ill medical and surgical patients: the Activity and Cognitive Therapy in ICU (ACT-ICU) trial.* Intensive Care Med, 2014. **40**(3): p. 370-9.

28. Needham, D.M., et al., *Early physical medicine and rehabilitation for patients with acute respiratory failure: a quality improvement project.* Arch Phys Med Rehabil, 2010. **91**(4): p. 536-42.

29. Davidson, J.E., et al., *Guidelines for Family-Centered Care in the Neonatal, Pediatric, and Adult ICU.* Crit Care Med, 2017. **45**(1): p. 103-128.

30. Nassar Junior, A.P., et al., *Flexible Versus Restrictive Visiting Policies in ICUs: A Systematic Review and Meta-Analysis.* Crit Care Med, 2018. **46**(7): p. 1175-1180.

31. Honiden, S. and M.D. Siegel, *Analytic Reviews: Managing the Agitated Patient in the ICU: Sedation, Analgesia, and Neuromuscular Blockade.* Journal of Intensive Care Medicine, 2010. **25**(4): p. 187-204.

32. Azimaraghi, O., et al., *Agitated Patients in the Intensive Care Unit: Guidelines for Causal Rather Than Symptomatic Treatment are Warranted.* Journal of Intensive Care Medicine, 2023. **38**(2): p. 238-240.

33. Aubanel, S., et al., *Therapeutic options for agitation in the intensive care unit.* Anaesth Crit Care Pain Med, 2020. **39**(5): p. 639-646.

34. Cohen, I.L., et al., *Management of the agitated intensive care unit patient.* Critical Care Medicine, 2002. **30**(1): p. S97-S123.
