# Supplementary material for: A multicomponent intervention program to Prevent and Reduce AgItation and phySical rEstraint use in the ICU (PRAISE): study protocol for a multicenter, stepped-wedge, cluster randomized controlled trial
Source: Trials. 2023 Dec 11;24:800. doi: 10.1186/s13063-023-07807-x (PMC10712112; doi:10.1186/s13063-023-07807-x)
Supplement: Supplementary file 3 — Additional file 3. Consent form patient. [file 13063_2023_7807_MOESM3_ESM.docx]

**Toestemmingsverklaring patiënt**

Voor deelname aan het wetenschappelijk onderzoek:

| **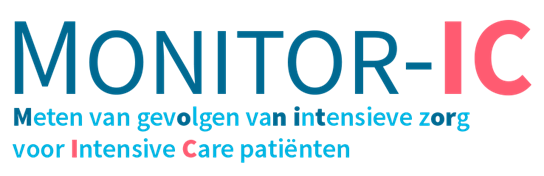** |
| --- |

- Ik ben naar tevredenheid over het onderzoek MONITOR-IC geïnformeerd en ik weet dat het Radboudumc voor de verwerking van mijn persoonsgegevens voor dit onderzoek de verantwoordelijke is in de zin van de Algemene verordening gegevensbescherming (AVG).
- Ik heb de informatie over het MONITOR-IC onderzoek goed gelezen (informatiebrief en het privacyreglement op de website [www.monitor-ic.nl](file:///\\UMCFS080\ICdata$\Monitor-IC\Kostenonderzoek\Vektis\DocumentenTijmen\Aangepast%20nav%20Feedback%20Tijmen\www.monitor-ic.nl)) en ik weet dat ik in het privacyreglement o.a. kan terugvinden welke soorten persoonsgegevens precies worden verzameld en verwerkt voor wetenschappelijk onderzoek.
- Ik ben voldoende in de gelegenheid gesteld om vragen over het onder­zoek te stellen. Mijn eventuele vragen zijn naar tevredenheid beantwoord en ik weet dat ik in het privacyreglement kan lezen waar ik terecht kan als ik weer vragen heb.
- Ik heb goed over deelname aan het onder­zoek kunnen nadenken.
- Ik weet dat meedoen geheel vrijwillig is en dat, of ik wel of geen toestemming geef, dit geen enkele invloed heeft op de behandeling en zorg die ik van mijn zorgverlener(s) ontvang of nodig heb.
- Ik weet dat de verzamelde gegevens worden bewaard voor tenminste 15 jaar, zoals beschreven in het privacydocument.
- Ik heb het recht mijn toestemming op ieder moment weer in te trekken, zonder daarvoor een reden op te geven. Ik weet dat ik in het privacyreglement kan lezen waar ik terecht kan om mijn deelname aan dit onderzoek te beëindigen.

| Ik geef toestemming | | |
| --- | --- | --- |
|  | Ja | Nee |
| 1) Ik geef toestemming voor deelname aan het MONITOR-IC onderzoek en ontvang daartoe op gezette tijden vragenlijsten. |  |  |
| 2) Ik geef eveneens toestemming om de relevante soorten gegevens, als weergegeven in het privacyreglement bijlage I, uit mijn ziekenhuisdossier voor uitsluitend dit onderzoek te gebruiken. |  |  |
| 3) Ik geef eveneens toestemming om de relevante soorten gegevens, als weergegeven in het privacyreglement bijlage I, bij de zorgverzekeraar/zorgkantoor (v.w.b. langdurige zorg) waar ik ben verzekerd op te vragen en uitsluitend voor dit onderzoek te gebruiken. |  |  |

* Punt 1 en 2 zijn minimaal nodig (toestemming ‘Ja’) voor de vrijwillige deelname aan de MONITOR-IC

Indien u niet wilt deelnemen, dan graag bij alle punten ‘Nee’ aankruisen.

|  | *(gebruikt u a.u.b. blokletters)* | |
| --- | --- | --- |
| Voorletters: |  | |
| Achternaam (en achternaam partner): |  | |
| Geboortedatum: | - - |  |
| E-mailadres: |  | |
|  |  | |

Datum ondertekening: ….…..…- …….……- 20…..…..

*U kunt dit formulier terugsturen naar het ziekenhuis waar u bent opgenomen. U kunt hiervoor gebruik maken van de kleine retour antwoordenvelop. U hoeft geen postzegel te plakken.*

**Handtekening**
