# Supplementary material for: A multicomponent intervention program to Prevent and Reduce AgItation and phySical rEstraint use in the ICU (PRAISE): study protocol for a multicenter, stepped-wedge, cluster randomized controlled trial
Source: Trials. 2023 Dec 11;24:800. doi: 10.1186/s13063-023-07807-x (PMC10712112; doi:10.1186/s13063-023-07807-x)
Supplement: Supplementary file 4 — Additional file 4. Consent form legal representative. [file 13063_2023_7807_MOESM4_ESM.docx]

**Toestemmingsverklaring wettelijk vertegenwoordiger**

Als wettelijk vertegenwoordiger van de hierna genoemde deelnemer ben ik gevraagd om namens hem/haar toestemming te geven voor deelname aan het wetenschappelijk onderzoek:

| **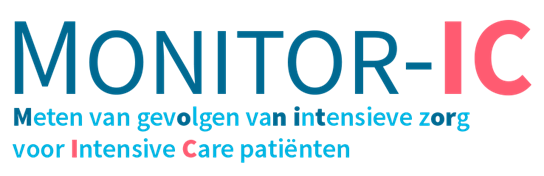** |
| --- |

*Gegevens deelnemende patiënt*

Achternaam (en achternaam partner): ……………………………………………………….

Voorletter(s): ……………………………………………………….

Geboortedatum: │__│__│-│__│__│-│__│__│­__│__│ (dd-mm-jjjj)

Emailadres patiënt: ………………………………………………………

- Ik ben naar tevredenheid over het onderzoek MONITOR-IC geïnformeerd en ik weet dat het Radboudumc voor de verwerking van de persoonsgegevens van de deelnemer aan dit onderzoek en mijn persoonsgegevens de verantwoordelijke is in de zin van de Algemene verordening gegevensbescherming (AVG).
- Ik heb de informatie over het MONITOR-IC onderzoek goed gelezen (informatiebrief en het privacyreglement op de website [www.monitor-ic.nl](http://www.monitor-ic.nl)) en ik weet dat ik in het privacyreglement o.a. kan terugvinden welke soorten persoonsgegevens worden verzameld en verwerkt voor wetenschappelijk onderzoek.
- Ik ben voldoende in de gelegenheid gesteld om vragen over het onderzoek te stellen. Mijn eventuele vragen zijn naar tevredenheid beantwoord en ik weet dat ik in het privacyreglement kan lezen waar ik terecht kan als ik weer vragen heb.
- Ik heb goed over deelname van deze persoon aan het onderzoek kunnen nadenken.
- Ik weet dat het meedoen geheel vrijwillig is en dat, of ik wel of geen toestemming geef, dit geen enkele invloed heeft op de behandeling en zorg die mijn naaste van zijn/haar zorgverlener(s) ontvangt of nodig heeft.
- Ik weet dat de verzamelde gegevens worden bewaard voor tenminste 15 jaar.
- Ik weet dat ik/mijn naaste het recht heb(ben) om op ieder moment de deelname in te trekken, zonder daarvoor een reden op te geven. Ik weet dat ik in het privacyreglement kan lezen waar ik terecht kan om deelname van deze persoon aan dit onderzoek te beëindigen.
- Ik weet dat mijn naaste, indien hij/zij daartoe in staat is, zelf ook over de deelname zal worden geïnformeerd en een door mij toegezegde deelname, dan te allen tijde kan heroverwegen.
- Ik weet dat de persoonlijke gegevens die hieronder invul, opgenomen worden in de database.

| Ik geef toestemming, | | |
| --- | --- | --- |
|  | Ja | Nee |
| 1) Ik geef toestemming voor deelname van mijn naaste aan het MONITOR-IC onderzoek, tot het intrekken van toestemming, en ontvang daartoe op gezette tijden vragenlijsten voor mijn naaste. |  |  |
| 2) Ik geef eveneens toestemming om de relevante soorten gegevens van mijn naaste, als weergegeven in het privacyreglement bijlage I, uit het ziekenhuisdossier voor uitsluitend dit onderzoek te gebruiken. |  |  |
| 3) Ik geef eveneens toestemming om de relevante soorten gegevens van mijn naaste, als weergegeven in het privacyreglement bijlage I, bij de zorgverzekeraar/zorgkantoor (v.w.b. langdurige zorg) waar hij/zij is verzekerd, op te vragen en uitsluitend voor dit onderzoek te gebruiken. |  |  |

* Punt 1 en 2 zijn minimaal nodig (toestemming JA) voor de vrijwillige deelname aan de MONITOR-IC

Indien u niet wilt deelnemen, dan graag bij alle punten ‘Nee’ aankruisen.

*Uw gegevens* (wettelijk vertegenwoordiger)

|  | *(graag in blokletters)* |
| --- | --- |
| Voorletters: |  |
| Achternaam: |  |
| E-mailadres: |  |
| Relatie tot de deelnemer: |  |
| **Handtekening** |  |

Datum ondertekening: ….…..…- …….……- 20…..…..

*U kunt dit formulier terugsturen naar het ziekenhuis waar de deelnemers is opgenomen. U kunt hiervoor gebruik maken van de kleine retour antwoordenvelop. U hoeft geen postzegel te plakken.*

Indien de vragenlijst naar een ander adres moet worden verstuurd, vult u hier het postadres in:

| Adres: |  |
| --- | --- |
| Postcode/Plaatsnaam: |  |
|  |  |
