# Supplementary material for: A multicomponent intervention program to Prevent and Reduce AgItation and phySical rEstraint use in the ICU (PRAISE): study protocol for a multicenter, stepped-wedge, cluster randomized controlled trial
Source: Trials. 2023 Dec 11;24:800. doi: 10.1186/s13063-023-07807-x (PMC10712112; doi:10.1186/s13063-023-07807-x)
Supplement: Supplementary file 5 — Additional file 5. Ethical approval (translated). [file 13063_2023_7807_MOESM5_ESM.docx]

**Title of the research protocol: Prevention of patient’s agitation and enhancement of their safety (PRAISE)”**

**File number METC East Netherlands: 2022-16133**

**Principal Investigator's Name: Bram Tilburgs**

**Research Center Name: Radboudumc**

**Applicant's Name: Rens Kooken**

**Submission Date: January 10, 2023**

**Panama Number: 114163**

Dear Rens Kooken,

You have requested the committee to determine whether the above-mentioned study falls under the Medical Research Involving Human Subjects Act (WMO) and, as a result, needs to be assessed by an accredited medical ethics review committee.

The research participants are not subjected to WMO-obligatory procedures, and no WMO-obligatory behaviors are imposed upon them.

Based on this, the committee declares that the research does not fall under the WMO. Therefore, a positive assessment from the METC East Netherlands or any other accredited medical ethics review committee is not required for its execution. The committee has only assessed your research in terms of WMO obligation and has not subjected it to substantive review (hence, the subject information for participants cannot state that the research has been approved by an METC).

This decision has been reached after reviewing the following documents:

- Cover letter dated January 10, 2023
- Research protocol version 1 dated January 6, 2023
- Informed consent form for legal representative version 14 dated January 2023
- Informed consent form for the patient version 14 dated January 2023
- Information letter version 10 received on January 10, 2023
- Questionnaire received on January 10, 2023

If you haven't already done so, I recommend checking with the participating centers whether the execution of your non-WMO research requires an assessment by the local non-WMO review committee (for Radboudumc, please refer to the METC East Netherlands website for non-WMO research obligations).

I would also like to draw your attention to the Integrated Quality System for Scientific Research (IKS) for laws and regulations and Radboudumc's policy regarding non-WMO research. Under the Radboudumc SOPs section, you will find the 'Normenkader statusonderzoek' (Framework for status research). If you have any questions regarding the information in the IKS, you can contact the RTC Clinical Studies (they will discuss your question(s) with experts in Radboudumc and provide advice).

I trust this message has been of assistance to you.

Best regards,

Prof. Dr. Gerard Rongen, Chairman / MREC Chair

**METC East Netherlands**

[METCoost-en-CMO@radboudumc.nl](mailto:METCoost-en-CMO@radboudumc.nl)

T (024) 3613154

**Radboud University Medical Centre**

Dentistry Building

Philips van Leydenlaan 25 (route 348), Nijmegen

[www.radboudumc.nl](http://www.radboudumc.nl)

[www.metc-oost-nederland.nl](http://www.metc-oost-nederland.nl)

cc RTCCS

------------------------------------------------------------------------------------------------------------------------------------

**Title: PRevention of pAtient’s agItation and enhancement of their SafEty (PRAISE): improving intensive care treatment using a multicomponent pharmacological intervention**

**Filenumber: 2022-16133**

Dear Rens Kooken ,

Please be informed that the abovementioned study will be carried out in accordance with the applicable legislation concerning reviewal by an accredited research ethics committee such as Medical Research involving Human Subjects Act and Medical Treatment Contracts Act.

Best regards,

Prof. dr. Gerard Rongen, MREC chair

Research Ethics Committee

Radboud University Medical Centre
