# Supplementary material for: A multicomponent intervention program to Prevent and Reduce AgItation and phySical rEstraint use in the ICU (PRAISE): study protocol for a multicenter, stepped-wedge, cluster randomized controlled trial
Source: Trials. 2023 Dec 11;24:800. doi: 10.1186/s13063-023-07807-x (PMC10712112; doi:10.1186/s13063-023-07807-x)
Supplement: Supplementary file 6 — Additional file 6. Funding documentation. [file 13063_2023_7807_MOESM6_ESM.docx]

Dear Mr. Smit,

We are pleased to inform you that ZonMw's board has decided to grant your subsidy application titled "PRevention of pAtent’s agItation and enhancement of theIr SafEty (PRAISE): improving intensive care using a multi-component pharmacological intervention." In this letter, you will find more information about the rationale behind this decision and the obligations you must fulfill before your project can commence. Attachment 1 contains additional obligations that apply to you as the subsidy recipient.

**Subsidy Amount and Project Duration**

You will receive a subsidy of up to €494,500 for a maximum duration of 48 months. Of this amount, €5,000 has been allocated for Open Access Publication Costs, including any applicable VAT.

**Basis for the Decision**

All subsidy applications were evaluated for relevance and quality as outlined in the subsidy call "Goed Gebruik Geneesmiddelen - Complexe Interventies Ronde 3" (Good Use of Medicines - Complex Interventions Round 3). The program committee of the Good Use of Medicines program provides a positive recommendation for your subsidy application. ZonMw considers the committee's advice to have been carefully considered, and we have based our decision on it.

*Relevance Assessment*

The committee has provided the following assessment regarding the relevance of your subsidy application: relevant. The assessment takes into account the input from the patient panel of the Dutch Patients Federation (Patiëntenfederatie Nederland). The relevance assessment is based on the following arguments:

The committee believes that the topic of your proposal is relevant and aligns well with the objectives of the Good Use of Medicines program. The project, through a combination of dexmedetomidine and non-pharmacological interventions, has the potential to lead to improved outcomes in patients, along with potential cost savings for healthcare.

The committee, however, finds the estimated potential cost savings to be somewhat optimistic and is not entirely convinced about the guaranteed dissemination of the results. They request that you pay sufficient attention to this aspect during the study.

*Quality Assessment*

The committee has provided the following assessment regarding the quality of your subsidy application: good. This assessment is based on your subsidy application, evaluations by reviewers, input from the patient panel, and your responses.

The quality assessment is based on the following arguments:

The committee believes that your project has a clear problem statement, and its objectives and chosen design align well with it.

Reviewers 3395073 and 3395083 have raised questions about the inclusion procedure. The committee finds your response on this point during the consultation phase not entirely clear and requests that you make a comprehensive effort to include as many participants as possible during the study. It is essential to document which patients are included and excluded, and the reasons for exclusion (e.g., no risk of agitation, no informed consent provided, exclusion due to other reasons such as ICU workload).

The committee finds the timing of outcome measurements, 12 months after admission, to be late and recommends measuring outcomes (also) at 6 months.

*Summary Judgment*

Based on the final assessment of the relevance and quality of your subsidy application, your request can be granted.

**Project Commencement**

Before your project can commence, you must meet the obligations outlined below:

Within 4 weeks of the date of this letter:

- A task will be available in "Mijn ZonMw" to complete the notification form.

*Open Science - Data Management*

To ensure that data from your project remains reusable in the future, you must create a data management plan. Information on how to do this can be found on the ZonMw website. Follow the steps outlined in the procedure starting from this link. Submit your data management plan and (preliminary) core data before the start date of your project. Both can be sent to geneesmiddelen@zonmw.nl. If you are not collecting data, please inform the program team.

*Declarations and Permits*

Before the start of your project, you must comply with all requirements for conducting the research. We recommend initiating any necessary procedures in a timely manner. This may include obtaining a positive assessment from an accredited medical ethics review committee (METC), approval from the Central Committee on Research Involving Human Subjects (CCMO), a project permit from the Central Committee for Animal Testing (CCD), or a permit under the Population Screening Act (WBO). You can inquire with the relevant authorities to determine if your project requires such declarations or permits.

ZonMw would like to draw your attention to the new EU Regulation on Clinical Trials (CTR), which has been in effect since January 31, 2022. More information can be found on the CCMO website.

**First Installment**

Once you have fulfilled all the obligations for the start of your project, you will receive a notification about the advance payment for your project, and you will receive the first installment.

**Questions**

If you have any questions, please contact your contact person, Esther Dalhuisen-Suurland, via email at geneesmiddelen@zonmw.nl or by phone at 070 349 54 64. Please have your dossier number on hand for prompt assistance. Your dossier number is: 10140302110007.

**Objection or Complaint**

If you disagree with this decision, you have the option to submit an objection. If you are considering this, we advise you to first contact your designated contact person. An objection must be sent to the ZonMw Board, Attention: ZonMw Complaints Committee, P.O. Box 93 245, 2509 AE The Hague, within 6 weeks of the date of this letter. If you are dissatisfied with how ZonMw has handled your application, you can inform us or file a complaint. More information can be found on the ZonMw website at www.zonmw.nl/signalerenklagenbezwaarmaken.

Congratulations on the approval of your subsidy application, and we wish you success in carrying out your project!

Sincerely,

On behalf of ZonMw's Board,

Hannie Bonink

Program Director

A copy will be sent via email to:

RadboudUMC, Dr. B. Tilburgs PhD
